# A thermal performance curve perspective explains decades of disagreements over how air temperature affects the flight metabolism of honey bees

**DOI:** 10.1242/jeb.246926

**Published:** 2024-04-08

**Authors:** Jordan R. Glass, Jon F. Harrison

**Affiliations:** ^1^ Department of Zoology and Physiology, University of Wyoming, Laramie, WY 82071, USA; ^2^ School of Life Sciences, Arizona State University, AZ 85281, USA

**Keywords:** Flight muscle temperature, Thermoregulation, Wing kinematics, Metabolic rate, *Apis mellifera*

## Abstract

While multiple studies have shown that honey bees and some other flying insects lower their flight metabolic rates when flying at high air temperatures, critics have suggested such patterns result from poor experimental methods as, theoretically, air temperature should not appreciably affect aerodynamic force requirements. Here, we show that apparently contradictory studies can be reconciled by considering the thermal performance curve of flight muscle. We show that prior studies that found no effects of air temperature on flight metabolism of honey bees achieved flight muscle temperatures that were near or on equal, opposite sides of the thermal performance curve. Honey bees vary their wing kinematics and metabolic heat production to thermoregulate, and how air temperature affects the flight metabolic rate of honey bees is predictable using a non-linear thermal performance perspective of honey bee flight muscle.

## Teaser

**Highlighted Article:** Decades of discrepancies and disagreements regarding whether air temperature affects the flight metabolism of honey bees can be reconciled by considering the thermal performance curve of flight muscle.

## INTRODUCTION

Whether honey bees vary flight metabolism when flying across a range of air temperatures remains controversial. Several studies found that flight metabolic rates of honey bees decrease at high air temperatures (Harrison et al., 1996a,b; Roberts and Harrison, 1999) and that flight metabolism increases with temperature across low-temperature ranges (Harrison et al., 2001). In contrast, Heinrich (1980) and Woods et al. (2005) found that air temperature had no effect on the flight metabolism of unloaded honey bees (i.e. carrying no pollen or nectar). Woods and colleagues (2005) further suggested that flight metabolic rates are independent of air temperature if investigators are careful to ensure continuous flight with natural light stimuli. In an attempt to explain these discrepancies, Harrison and Fewell (2002) suggested that the effects of air temperature on flight metabolic rate may depend on how the flight muscle temperatures tested relate to the optimal temperature for flight muscle function. They proposed that we would only see a decline in flight metabolic rate at higher temperatures if the air temperatures tested push flight muscle temperatures into this above-optimum range. However, at the time, no studies had measured a thermal performance curve for flight metabolism for any endothermic insects, so this conjecture could not be tested. In the present study, and in our recent studies (Glass and Harrison, 2022; Glass et al., 2024), we address this decades-old controversy.

Harrison and colleagues (1996a,b) found that honey bees decrease wingbeat frequency as well as flight metabolic rate in response to high air temperatures. However, critics raised doubts that wingbeat frequency declines with air temperature (Heinrich and Esch, 1997), pointing out that the aerodynamic force requirements, and thus work, for animal flight are nearly independent of air temperature (Ellington, 1984; Dudley, 2000). In response to these criticisms, Roberts and Harrison (1999) hypothesized that honey bees decrease their wingbeat frequency to reduce metabolic heat production when flying in the heat while adjusting other kinematic contributions, such as increasing their stroke amplitude, potentially allowing honey bees to fly more efficiently when things get hot. Until recently, no study had measured the metabolic and kinematic responses of honey bees flying at high air and flight muscle temperatures (Glass et al., 2024). We now know that honey bees flying at 40°C air temperature can lower their flight metabolism by decreasing their wingbeat frequency by about 10% and increasing their stroke amplitude by the same amount, allowing hot bees to generate the same amount of aerodynamic force as bees flying in cooler conditions (Glass et al., 2024). With this knowledge, we can begin to reconcile the differences between these studies in light of the thermal performance curve for flight metabolism in honey bees (Glass and Harrison, 2022).

**Fig. 1.**
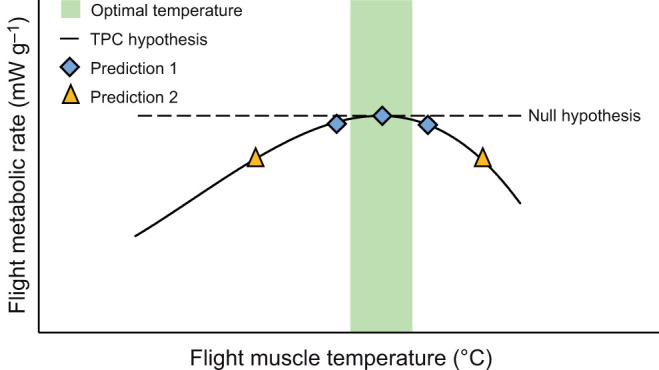
**Contrasting hypotheses and predictions of the effects of flight muscle temperature on the flight metabolic rates of free-flying honey bees.** The thermal performance curve hypothesis (TPC hypothesis) proposes that the thermal performance curve of flight muscle determines the effect of air temperature on flight metabolic rate. Studies testing flight muscle temperatures close to optimal (prediction 1) and those testing air temperatures that set flight muscle temperatures equally above and below the optimum (prediction 2) will not observe air temperature effects on flight metabolic rate. The null hypothesis is that flight metabolism is independent of air temperature.

Here, we test the hypothesis, which we will call the thermal performance curve hypothesis (or TPC hypothesis) that the thermal performance curve of flight muscle determines the effect of air temperature on flight metabolic rates of honey bees. By measuring the maximal force production (Coelho, 1991a) and maximal metabolic power production of honey bees (Glass and Harrison, 2022), we know the optimal temperature (circa 39°C), and the shape of the thermal performance curve for honey bee flight muscle. Based on the TPC hypothesis, we predict that unloaded honey bees with flight muscle temperatures below or above this optimum will show increasingly depressed flight metabolic rates (Fig. 1 – ‘TPC hypothesis’). We also predict that studies that test flight muscle temperatures in a narrow range close to optimal will not observe air temperature effects on flight metabolic rate (Fig. 1 – ‘Prediction 1’). Furthermore, we predict that studies that test air temperatures that set flight muscle temperatures similarly above and below the optimum will also not observe air temperature effects on flight metabolism (Fig. 1 – ‘Prediction 2’). In contrast, the null hypothesis is that the flight metabolism of honey bees is independent of air temperature (Fig. 1 – ‘Null hypothesis’) and set by the biomechanical requirements of hovering flight, which are relatively independent of temperature (Ellington, 1984; Dudley, 2000). Under this null hypothesis, we predict no change in flight metabolism across a wide range of flight muscle temperatures.

To experimentally assess the effect of air and flight muscle temperature on flight metabolic rate, we used previously published data (Heinrich, 1980; Harrison et al., 2001; Woods et al., 2005; Glass and Harrison, 2022), as well as new data we report here. The thermal performance curve for maximal flight metabolism was calculated by averaging the maximal flight metabolic rate for all honey bees within a 0.5°C range of flight muscle temperatures, plotted across the full range of flight muscle temperatures (Glass and Harrison, 2022). Our data suggest that unloaded honey bees vary their flight metabolic rate to thermoregulate, and that differences among prior studies in the flight muscle temperatures achieved explain discrepancies in how air temperature affects flight metabolism in *Apis mellifera*.

## MATERIALS AND METHODS

### Study animals and location

The data in this study come from published work (i.e. Heinrich, 1980; Harrison et al., 2001; Woods et al., 2005; Glass and Harrison, 2022) and a new dataset presented here, which used the honey bee, *Apis mellifera* Linnaeus 1758. We digitally extracted data from the figures of the Heinrich (1980), Harrison et al. (2001) and Woods et al. (2005) studies, as these datasets were not accessible. Methods for these published studies can be found in each respective published article, but we briefly describe the experimental protocol for each below. From these previously published studies, we report the free-flying, mass-specific metabolic rates (mW g^−1^) of unloaded bees and their achieved flight muscle temperatures (°C) when flown at different air temperatures. We only included unloaded bees weighing <98 mg in this analysis, a delineation based on extrapolated values from Woods et al. (2005) (i.e. 90±8.8 mg, mean±s.d., *n*=78). By only including bees that weighed <100 mg, we also ensured the exclusion of bees transitioning from the role of brood care to foraging, which still carry significant hindgut content that can affect flight performance and metabolism (Glass et al., 2021; Glass and Harrison, 2022).

### Data standardization

Data from Glass and Harrison (2022) represent maximal aerobic performance for unloaded flying honey bees (see below for a brief description of these methods). These maximal aerobic performance estimates came from measurements of flight metabolism in which we flew honey bees in decreasing air densities. Flight in low-density air requires bees to generate higher mechanical power, eliciting maximal aerobic performance in the lowest density air (25–40% higher metabolic rates than for bees flown at normal air density; Roberts et al., 2004). We were confident these measures represented maximal performance because many of the tested bees failed to fly at lower air densities (Glass and Harrison, 2022). From the Harrison et al. (2001) study, we only included data of ‘winter bees’ flying in normoxic air (21% O_2_, 79% N_2_) at 24°C air temperature. Winter bees are specialized, long-lived honey bee workers that spend most of the winter within the hive (Kunert and Crailsheim, 1988; Kunc et al., 2019; Lee et al., 2022); they were collected from the hive mid-winter and forced to fly in the lab (Harrison et al., 2001). The Woods et al. (2005) data did not need to be standardized and are reported in the same units (i.e. mW g^−1^). Although Woods and colleagues (2005) measured the flight muscle temperatures of bees immediately after recording their flight metabolic rates, they did not include a single figure showing the relationship between flight metabolic rate and flight muscle temperature in their original study. To generate the Woods et al. (2005) data, we extracted their reported flight muscle temperatures (*n=*34) and mass-specific flight metabolic rates (*n*=19) for bees flying at different air temperatures and compared the air temperature at which each bee was flown. Using this approach, we were able to confidently link almost all the mass-specific flight metabolic rates with their corresponding muscle temperatures (*n=*18). Heinrich (1980) reported averaged flight metabolic rate values that he measured for 20 bees flown at 20 and 42°C air temperatures (*n=*10 per temperature), which we converted from ml O_2_ g^−1^ h^−1^ to mW g^−1^ for ease of comparison (see below for conversion coefficients), assuming simple carbohydrate catabolism (i.e. respiratory quotient=1.0; Beenakkers et al., 1984; Bertsch, 1984; Rothe and Nachtigall, 1989; Feuerbacher et al., 2003; Suarez et al., 2005). The new data reported here were measured as CO_2_ emission rates and were converted to mW g^−1^ using the same conversion coefficients as for the Heinrich (1980) data.

### Flight metabolic rate measurements

In this study, we captured outgoing foragers that were leaving the colony. After recording each bee's mass, we measured the CO_2_ production rates (i.e. an indirect measure of flight metabolism) of each bee inside a temperature-controlled room set to 20±0.5, 30±0.5 or 40±0.05°C air temperature (similar to Glass et al., 2024). We monitored the temperature inside the room using a thermocouple integrated with Expedata (Sable Systems International, Las Vegas, NV, USA). We used a random number generator (www.randomizer.org) to decide the order in which to sample from three available *A. mellifera* colonies maintained on the Tempe campus of Arizona State University.

We also measured the flight muscle temperatures of bees immediately after each metabolic measurement using a Physitemp model MT29/1 hypodermic microprobe (Clifton, NJ, USA; 29-gauge, time constant 0.025 s) and recorded flight muscle temperature data with a Pico Technology USB TC-08 Thermocouple Data Logger (St Neots, UK; see Glass et al., 2021; Glass and Harrison, 2022; Glass et al., 2024). Finally, we weighed the bee (±0.1 mg) using an A&D HR-120 Analytical Balance (Tokyo, Japan) and stored its body at −20°C.

Below are brief descriptions of the methods used in the other studies included in our analysis. For more information, please see the original studies.

#### Heinrich (1980) methods

Honey bees used in Heinrich's (1980) study were collected from the entrance of a single observation hive kept on the University of California, Berkeley campus, USA. To encourage bees to fly continuously, Heinrich removed each bee's tarsi (*n*=20 bees) so they could not cling to the sides of the sealed, glass flight metabolic chamber (volume 3.88 l), and he gently jostled and tilted the chamber to encourage any bees that had stopped flying during the trials. Heinrich ran his flight trials in a temperature-controlled room (maintained at either 20 or 42°C), giving the flight metabolic chamber enough time to reach thermal equilibrium before placing a bee inside. The flight metabolic chamber was made air-tight once the bee was placed inside, which was encouraged to fly for anywhere between 4.5 and 10 min. At the end of the flight trial, Heinrich took a sample of air from inside the sealed flight chamber using a syringe and injected the gas sample into an oxygen analyzer (Beckman E-2 paramagnetic oxygen analyzer). To measure body temperature, Heinrich flew a different set of tarsi-removed bees (*n=*34) for 3 min before recording body temperature with a hypodermic thermocouple.

#### Harrison et al. (2001) winter bee methods

Harrison et al. (2001) ran their experiment using honey bees collected directly from inside a single hive during mid-winter (January) in State College, Pennsylvania, USA. The researchers used gas cylinders of oxygen and nitrogen and a mass-flow controller to create a gas mixture of 79% N_2_, 21% O_2_, which flowed (at 2 l min^−1^) through a 300 ml flight metabolic chamber, then to a carbon dioxide analyzer (LI-COR 6252) in a differential, flow-through respirometry setup. They ran their flight metabolic trials in a temperature-controlled room that they kept at 24°C. Harrison and colleagues flew their test bees for 1.5 min, but they only averaged the carbon dioxide production rate over the last minute of the flight metabolic trial. Immediately after measuring carbon dioxide production, the researchers shook the bee into a bag and measured its flight muscle temperature with a hypodermic thermocouple.

#### Woods et al. (2005) methods

Woods et al. (2005) ran the experiment using one of the co-author's (Bernd Heinrich) personal honey bee hives in Hinesburg, VT, USA. The researchers measured the rate of carbon dioxide production of honey bees (*n=*19) flying across a range of air temperatures (18–39°C) by flowing dry, CO_2_-free air (at 860 ml min^−1^) through a differential, open-flow respirometry setup (LI-COR 6262 CO_2_ analyzer). The metabolic chamber itself was kept inside a shaded, outdoor, temperature-controlled cabinet during measurements. Woods and colleagues used a 500 ml Erlenmeyer flask as a flight metabolic chamber with a hypodermic thermocouple inserted through the lid to monitor the temperature of the flight chamber. To keep bees flying as continuously as possible, Woods and colleagues lightly shook or tapped the flight chamber when needed. The researchers flew each bee for 5 min during the metabolic trial, but it is unclear whether they averaged each bee's carbon dioxide production rate over the whole trial, or over a shorter period. After the flight metabolic trial, they shook the bee into a plastic bag and measured its body temperature with a hypodermic thermocouple within 10 s after the bee had stopped flying.

#### Glass and Harrison (2022) methods

Glass and Harrison (2022) ran experiments using three hives of honey bees maintained at Arizona State University, Tempe, USA. The researchers used gas cylinders of oxygen, nitrogen and helium and an 8-channell mass-flow controller (Flow-Bar 8, Sable Systems International) to create variable-density gas mixtures (range 0.441–1.288 kg m^−3^) to elicit maximal flight performance. Glass and Harrison measured the carbon dioxide production rate of flying honey bees using a differential, flow-through respirometry setup. After scrubbing these gas mixtures of CO_2_ and water, these variable-density gases flowed through the CO_2_ analyzer (LI-COR 6252), then through the 350 ml flight metabolic chamber (at 2 l min^−1^), and then back to the analyzer. They ran their flight metabolic trials in a temperature-controlled room that they kept at either 23 or 35°C. Glass and Harrison flew their test bees for 2 min. Immediately after measuring carbon dioxide production, the researchers shook the bee into a bag and measured its flight muscle temperature with a hypodermic thermocouple.

## RESULTS AND DISCUSSION

**Fig. 2.**
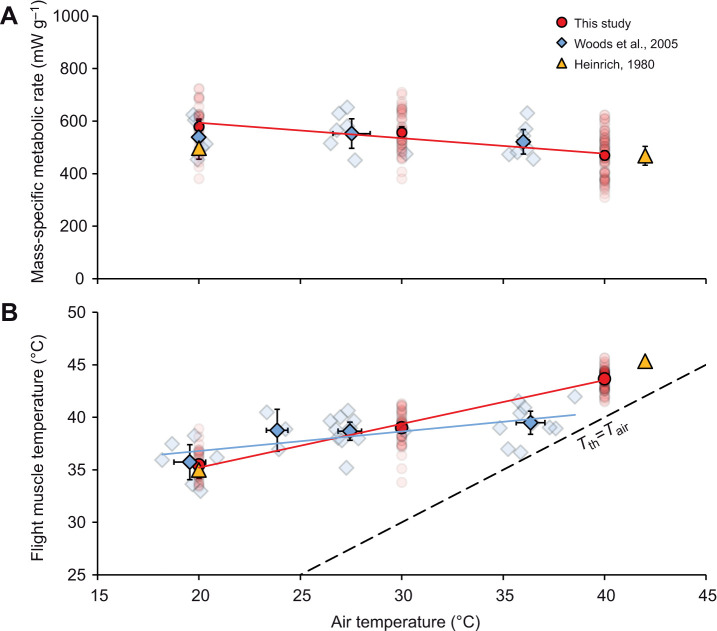
**Effect of air temperature on flight metabolic rate and flight muscle temperature.** (A) Heinrich (1980) and Woods et al. (2005) found no significant effect of air temperature on mass-specific flight metabolism (Heinrich, 1980: reported no difference; Woods et al., 2005: *y*=−1.675*x*+584.9, *n*=19, *R*^2^=0.03, *P*=0.51). However, like prior studies in our lab, we found that flight metabolic rate declined with air temperature (*y*=−5.883*x*+711.44, *n*=160, *R*^2^=0.27, *P*<0.001). (B) Higher air temperature increased flight muscle temperature of freely flying honey bees (Heinrich, 1980: only reported values as the mean±s.d.; Woods et al., 2005: *y*=0.181*x*+33.45, *n*=19, *R*^2^=0.27, *P*<0.01; this study: *y*=0.416*x*+26.88, *n*=160, *R*^2^=0.87, *P*<0.001). Fitted solid regression lines denote significance. The dashed line visualizes the slope of the line if the flight muscles of flying honey bees matched air temperature (*T*_th_=*T*_air_). In these figures, each solid point from this study and those of Heinrich (1980) and Woods et al. (2005) represent the mean±95% confidence limit (CL). The translucent points are included to show the distribution of the data from each study. The Heinrich (1980) study shows no data distribution because he only reported his values as the mean±s.e.m.

Data for summer-caught honey bees flying freely at different air temperatures from Heinrich (1980) and from Woods et al. (2005) suggest that flight metabolism is independent of air temperature (Fig. 2A). In contrast, the data presented in this study show a negative effect, with flight metabolic rates decreasing as the bees fly at higher air temperatures (Fig. 2A).

The data presented here and those reported by Heinrich (1980) and Woods et al. (2005) all show that the temperature of flight muscles of honey bees increases with increasing air temperature (Fig. 2B). However, the extent to which flight muscle temperature increases with air temperature differed among these studies (‘study’×air temperature interaction – generalized linear model: *n*=190, d.f.=1, χ^2^=30.4, *P*<0.0001; Table S1).

**Fig. 3.**
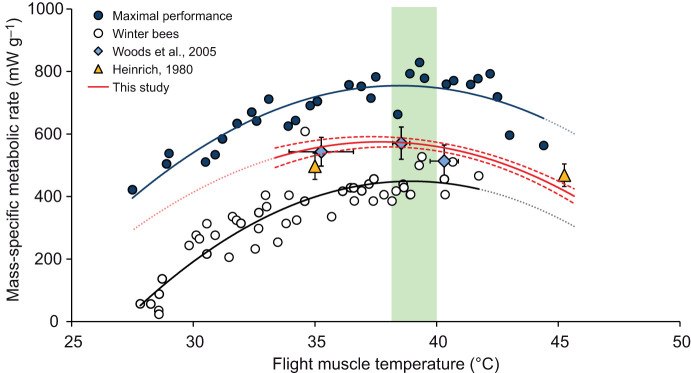
**Mass-specific flight metabolism of honey bees is asymmetrical, non-linear and strongly responds to changes in flight muscle temperature.** The shaded green area indicates the optimal temperature for force and metabolic power production (circa 39°C; Coelho, 1991a,b; Glass and Harrison, 2022). Points from the Heinrich (1980) study represent the mean±95% CL, as do those from the Woods et al. (2005) study, with the addition of horizontal 95% CL (Woods et al., 2005: linear fit to the raw data: *n=*18, *y*=−5.023*x*+730.66, *R*^2^=0.04, *P*=0.44). Fitted, solid polynomial lines represent significance [‘maximal performance’ (Glass and Harrison, 2022): *n*=30, *y*=−2.914*x*^2^+223.89*x*−3575.9, *R*^2^=0.79, *P*<0.001; ‘winter bees’ (Harrison et al., 2001): *n*=50, *y*=−3.213*x*^2^+250.29*x*–4425.7, *R*^2^=0.82, *P*<0.001]. The red regression line and the red dashed 95% CL lines are the polynomial fit for this dataset (*n*=160, *y*=−2.730*x*^2^+205.7*x*−3300.8, *R*^2^=0.27, *P*<0.001), with the points removed for clarity. The black and red dotted extension lines for each significant polynomial relationship are for visualization and are based on the fit of the data to each model.

Here, we show that the mass-specific metabolic response of flying honey bees to changes in flight muscle temperature is asymmetric and non-linear and that the relative change in response depends on the temperature of the bee relative to the optimal temperature for aerobic performance (Fig. 3; Tables S2, S3). The relatively narrow range of flight muscle temperatures achieved by honey bees measured by Woods and colleagues (2005) falls near the optimal temperature for aerobic metabolism (Glass and Harrison, 2022), showing no significant relationship between flight muscle temperature and flight metabolic rate of bees. Similarly, the flight metabolic rates reported by Heinrich (1980) lie well within the data reported here, showing an almost equidistant distribution on either side of the optimal temperature for flight metabolism.

The data presented in this study allow us to explain the contradictions observed in prior studies of air temperature effects on honey bee thermoregulation and flight metabolism. A key missing consideration is the differential effect air temperature had on the flight muscle temperatures of flying honey bees (Fig. 2; Table S1). Unlike our study and those of others (Heinrich, 1980; Harrison et al., 1996a,b; Roberts and Harrison, 1999), the bees from the Woods et al. (2005) study maintained their flight muscle temperatures relatively tightly near the optimal range across air temperatures. The confidence limits for our data plotting flight metabolic rate versus flight muscle temperature include most of the Woods et al. (2005) data as well as the data from Heinrich (1980). We conclude that a single, non-linear model, with an optimum and shape near to that for maximal flight metabolic rate, can predict the flight metabolic rates of unloaded honey bees as air temperature varies.

It is interesting that Woods et al. (2005) found such a low slope for flight muscle temperature on air temperature, suggesting outstanding thermoregulation – especially as they reported no change in metabolic heat production. Honey bees are thought to be unable to vary heat transfer to the abdomen, and major increases in evaporative heat loss only occur at higher air and muscle temperatures (Snodgrass, 1925; Heinrich, 1980; Coelho, 1991b; Roberts and Harrison, 1999). The slope we documented for honey bee flight muscle temperature on air temperature is similar to those documented by Heinrich (1980) and in a prior study from our lab (Roberts and Harrison, 1999). Thus, the outstanding thermoregulation in the Woods et al. (2005) study suggests an unknown thermoregulatory mechanism, relatively low statistical power in their study or technical issues in measuring flight muscle temperatures.

The various conclusions of the different studies on the effects of air temperature on flight metabolic rate can now be reconciled by considering the range of flight muscle temperatures achieved relative to the thermal performance curve of flight muscle. Harrison and colleagues (2001) found that ‘winter bees’ have relatively lower flight metabolic rates and achieve lower thoracic temperatures (Fig. 3). Winter bees are long-lived honey bee workers, with specialized physiology, that spend most of the winter within the hive (Kunert and Crailsheim, 1988; Kunc et al., 2019; Lee et al., 2022). As predicted by the thermal performance curve for flight metabolism (Glass and Harrison, 2022), flight metabolic rates of winter bees increased strongly with flight muscle temperatures across the lower muscle temperature ranges (Fig. 3). Even in winter bees with their low flight metabolic rates, flight metabolism peaked and plateaued near 39°C (Harrison et al., 2001; Fig. 3). Conversely, in the Harrison et al. (1996a,b) and Roberts and Harrison (1999) studies, which used summer bees, flight muscle temperatures ranged from 37 to 47°C. The flight muscle temperatures achieved in these studies fall mostly on the right side of the metabolic curve, resulting in decreasing flight metabolic rates as air temperatures increase. Finally, in the Woods et al. (2005) study, there was lower variation in thoracic temperatures (33–42°C), which centers these animals closer to the peak, optimal temperature, while in Heinrich's (1980) study, only two air temperatures were used (i.e. 20 and 42°C), resulting in flight muscle temperatures (i.e. 35 and 45°C) approximately equidistant from the optimal temperature for flight metabolism (i.e. 39°C; Glass and Harrison, 2022).

In this study, we show that decades of discrepancies and disagreements over whether air temperature affects the flight metabolism of honey bees have arisen from attempts to interpret data from incomplete perspectives. Together, these data suggest that the flight metabolic rate of honey bees reflects the asymmetric, non-linear thermal performance curve of flight muscle. It is interesting that this is so because, in theory, the flight metabolic rate for an unloaded, hovering bee need not track the maximal performance as temperature varies because an unloaded, hovering bee has a metabolic power output 20–30% below its metabolic capacity. The most obvious explanation is that bees fly with high frequency, low stroke amplitude flight kinematics at cool air temperatures to generate more metabolic heat and warm to near the optimal temperature, allowing them the option of higher maximal power output in the case of a large nectar load or a wind gust. At air temperatures that push flight muscle temperatures above optimum, switching to a lower frequency, higher amplitude, more efficient kinematic pattern allows them to minimize flight muscle elevation above optimum while preserving capacity for increasing performance (Glass et al., 2024).

## Supplementary Material

10.1242/jexbio.246926_sup1 Supplementary information

## References

[JEB246926C1] Beenakkers, A. T., Van der Horst, D. J. and Van Marrewijk, W. J. A. (1984). Insect flight muscle metabolism. *Insect Biochem.* 14, 243-260. 10.1016/0020-1790(84)90057-X

[JEB246926C2] Bertsch, A. (1984). Foraging in male bumblebees (*Bombus lucorum* L.): maximizing energy or minimizing water load? *Oecologia* 62, 325-336. 10.1007/BF00384264 28310885

[JEB246926C3] Coelho, J. R. (1991a). The effect of thorax temperature on force production during tethered flight in honeybee (*Apis mellifera*) drones, workers, and queens. *Physiol. Zool.* 64, 823-835. 10.1086/physzool.64.3.30158209

[JEB246926C4] Coelho, J. R. (1991b). Heat transfer and body temperature in honey bee (Hymenoptera: Apidae) drones and workers. *Environ. Entomol.* 20, 1627-1635. 10.1093/ee/20.6.1627

[JEB246926C5] Dudley, R. (2000). *The Biomechanics of Insect Flight*. Princeton, NJ: Princeton University Press.

[JEB246926C6] Ellington, C. P. (1984). The aerodynamics of hovering insect flight. IV. Aerodynamic mechanisms. *Philos. Trans. R. Soc. Lond. B Biol. Sci.* 305, 79-113. 10.1098/rstb.1984.0052

[JEB246926C7] Feuerbacher, E., Fewell, J. H., Roberts, S. P., Smith, E. F. and Harrison, J. F. (2003). Effects of load type (pollen or nectar) and load mass on hovering metabolic rate and mechanical power output in the honey bee *Apis mellifera*. *J. Exp. Biol.* 206, 1855-1865. 10.1242/jeb.00347 12728007

[JEB246926C8] Glass, J. R. and Harrison, J. F. (2022). The thermal performance curve for aerobic metabolism of a flying endotherm. *Proc. R. Soc. B* 289, 20220298. 10.1098/rspb.2022.0298 PMC9382204 35975442

[JEB246926C20] Glass, J. R. and Harrison, J. F. (2024). A thermal performance curve perspective explains decades of disagreements over how air temperature affects the flight metabolism of honey bees. Dryad. 10.5061/dryad.8w9ghx3vg PMC11058628 38487901

[JEB246926C9] Glass, J. R., Fisher, A. II, Fewell, J. H., DeGrandi-Hoffman, G., Ozturk, C. and Harrison, J. F. (2021). Consumption of field-realistic doses of a widely used mito-toxic fungicide reduces thorax mass but does not negatively impact flight capacities of the honey bee (*Apis mellifera*). *Environ. Pollut.* 274, 116533. 10.1016/j.envpol.2021.116533 33529906

[JEB246926C10] Glass, J. R., Burnett, N. P., Combes, S. A., Weisman, E., Helbling, A. and Harrison, J. F. (2024). Flying, nectar-loaded honey bees conserve water and improve heat tolerance by reducing wingbeat frequency and metabolic heat production. *Proc. Natl. Acad. Sci. USA* 121, e2311025121. 10.1073/pnas.2311025121 38227669 PMC10823226

[JEB246926C11] Harrison, J. F. and Fewell, J. H. (2002). Environmental and genetic influences on flight metabolic rate in the honey bee, *Apis mellifera*. *Comp. Biochem. Physiol. Part A: Mol. Integr. Physiol.* 133, 323-333. 10.1016/S1095-6433(02)00163-0 12208303

[JEB246926C12] Harrison, J. F., Fewell, J. H., Roberts, S. P. and Hall, H. G. (1996a). Achievement of thermal stability by varying metabolic heat production in flying honeybees. *Science* 274, 88-90. 10.1126/science.274.5284.88 8810252

[JEB246926C13] Harrison, J. F., Nielsen, D. I. and Page, R. E. Jr. (1996b). Malate dehydrogenase phenotype, temperature and colony effects on flight metabolic rate in the honey-bee, *Apis mellifera*. *Funct. Ecol.* 10, 81-88. 10.2307/2390265

[JEB246926C14] Harrison, J. F., Camazine, S., Marden, J. H., Kirkton, S. D., Rozo, A. and Yang, X. (2001). Mite not make it home: tracheal mites reduce the safety margin for oxygen delivery of flying honeybees. *J. Exp. Biol.* 204, 805-814. 10.1242/jeb.204.4.805 11171363

[JEB246926C15] Heinrich, B. (1980). Mechanisms of body-temperature regulation in honeybees, *Apis mellifera* II. Regulation of thoracic temperature at high air temperatures. *J. Exp. Biol.* 85, 73-87. 10.1242/jeb.85.1.73

[JEB246926C16] Heinrich, B. and Esch, H. (1997). Honeybee thermoregulation. *Science* 276, 1013-1013. 10.1126/science.276.5315.1013e 9173532

[JEB246926C17] Kunc, M., Dobeš, P., Hurychová, J., Vojtek, L., Poiani, S. B., Danihlík, J., Havlík, J., Titĕra, D. and Hyršl, P. (2019). The year of the honey bee (*Apis mellifera* L.) with respect to its physiology and immunity: a search for biochemical markers of longevity. *Insects* 10, 244. 10.3390/insects10080244 31394797 PMC6723739

[JEB246926C18] Kunert, K. and Crailsheim, K. (1988). Seasonal changes in carbohydrate, lipid and protein content in emerging worker honeybees and their mortality. *J. Apic. Res.* 27, 13-21. 10.1080/00218839.1988.11100775

[JEB246926C19] Lee, S., Kalcic, F., Duarte, I. F., Titĕra, D., Kamler, M., Mrna, P., Hyršl, P., Danihlík, J., Dobes, P., Kunc, M. et al. (2022). 1H NMR profiling of honey bee bodies revealed metabolic differences between summer and winter bees. *Insects* 13, 193. 10.3390/insects13020193 35206766 PMC8875373

[JEB246926C21] Roberts, S. P. and Harrison, J. F. (1999). Mechanisms of thermal stability during flight in the honeybee *Apis mellifera*. *J. Exp. Biol.* 202, 1523-1533. 10.1242/jeb.202.11.1523 10229698

[JEB246926C22] Roberts, S. P., Harrison, J. F. and Dudley, R. (2004). Allometry of kinematics and energetics in carpenter bees (*Xylocopa varipuncta*) hovering in variable-density gases. *J. Exp. Biol.* 207, 993-1004. 10.1242/jeb.00850 14766958

[JEB246926C23] Rothe, U. and Nachtigall, W. (1989). Flight of the honey bee: IV. Respiratory quotients and metabolic rates during sitting, walking and flying. *J. Comp. Physiol. B* 158, 739-749. 10.1007/BF00693012

[JEB246926C24] Snodgrass, R. E. (1925). *Anatomy and Physiology of the Honeybee*, Vol. 20. McGraw-Hill Book Company, Inc.

[JEB246926C25] Suarez, R. K., Darveau, C. A., Welch, K. C. Jr., O'Brien, D. M., Roubik, D. W. and Hochachka, P. W. (2005). Energy metabolism in orchid bee flight muscles: carbohydrate fuels all. *J. Exp. Biol.* 208, 3573-3579. 10.1242/jeb.01775 16155228

[JEB246926C26] Woods, W. A. Jr., Heinrich, B. and Stevenson, R. D. (2005). Honeybee flight metabolic rate: does it depend upon air temperature? *J. Exp. Biol.* 208, 1161-1173. 10.1242/jeb.01510 15767315

