# Supplementary material for: A thermal performance curve perspective explains decades of disagreements over how air temperature affects the flight metabolism of honey bees
Source: J Exp Biol. 2024 Apr 8;227(7):jeb246926. doi: 10.1242/jeb.246926 (PMC11058628; doi:10.1242/jeb.246926)
Supplement: Supplementary information [file jexbio-227-246926-s1.pdf]

Table S1. The linear fit, sample size, coefficient of determination, and the p-value for regressions depicting the relationship between mass-specific flight metabolism of honey bees and air temperature. Here, we give the statistical values reported by Woods *et al*. from their original study.

Study	Linear fit	*n*	R^2	*p*-value
This study	y = -5.883x + 711.44	160	0.27	< **0.001*****
Woods *et al*., 2005	y = -1.675x + 584.9	19	0.03	0.51
Heinrich, 1980*	y = -1.308x + 523.1	20	-	-

***Note: The linear fit equation for the data from Heinrich, 1980 is to show the general trend of the data and should not be considered significant as Heinrich only reported means ± S.D.**

Table S2. The linear fit, sample size, coefficient of determination, and the p-value for regressions depicting the relationship between flight muscle temperature of honey bees and air temperature. Here, we give the statistical values reported by Woods *et al*. from their original study.

Study	Linear fit	*n*	R^2	*p*-value
This study	y = 0.416x + 26.88	160	0.87	< **0.001*****
Woods *et al*., 2005	y = 0.181x + 33.35	32	0.27	< **0.01****
Heinrich, 1980*	y = 0.471x + 25.57	34	-	-

***Note: The linear fit equation for the data from Heinrich, 1980 is to show the general trend of the data and should not be considered significant as Heinrich only reported means ± S.D.**

Journal of Experimental Biology • Supplementary information

Table S3. The regression fit, sample size, coefficient of determination, and the p-value for regressions depicting the relationship between flight muscle temperature of mass-specific flight metabolic rate of honey bees. We also report the Akaike information criterion (AIC) for a linear and polynomial fit for each study. Bolded values denote which regression-type best fits each dataset. Here, we give the statistical outputs from our analysis of the Woods *et al*. data, which were digitally extracted from the figures of the original study.

Study	Regression fit	*n*	R^2	*p*-value	Linear AIC	Polynomial AIC
This study	$y = -2.730x^2 + 205.7x - 3300.8$	160	0.27	< **0.001***	1882.04	**1860.44**
Glass & Harrison, 2022	$y = -2.914x^2 + 223.89x - 3575.9$	30	0.79	< **0.001***	351.89	**324.98**
Harrison *et al*., 2001	$y = -3.213x^2 + 250.29x - 4425.7$	50	0.82	< **0.001***	577.14	**556.64**
Woods *et al*., 2005	$y = -5.023x + 730.66$	18	0.04	0.44	**204.95**	206.78
Heinrich, 1980*	$y = -2.808x + 595.21$	20	-	-	-	-

***Note: The linear fit equation for the Heinrich, 1980 data is to show the general trend of the data and should not be considered significant as Heinrich only reported means ± S.D.**
